# Synthesis, Characterization, and Application of Polymer-Based Materials

**DOI:** 10.3390/molecules30153244

**Published:** 2025-08-02

**Authors:** Zlatan Zlatev Denchev, Nadya Vasileva Dencheva

**Affiliations:** IPC—Institute for Polymers and Composites, School of Engineering, Campus of Azurém, University of Minho, 4800 Guimarães, Portugal

## 1. Introduction

The field of polymer-based materials is advancing rapidly, driven by the growing demand for sustainable technologies, enhanced functionalities, and cutting-edge manufacturing methods. This *Molecules* Special Issue, titled “Synthesis, Characterization, and Application of Polymer-Based Materials”, presents a selection of contributions—research and review articles—that reflect both recent developments and persistent challenges in this dynamic area of materials science. The first edition of this Special Issue was closed on 30 September 2024.

Comprising three review articles and eleven original research papers, this first edition covers a broad range of topics spanning from eco-friendly synthesis and hybrid polymer systems to machine learning–assisted material design and performance optimization. A total of 67 authors from research centers in China, the USA, Japan, Portugal, and Poland contributed to this collaborative effort.

## 2. Coverage of Emerging Trends

A prominent theme throughout the Special Issue is the development of *sustainable and environmentally conscious polymer synthesis* strategies. For instance, Agbo et al. [Contribution 1] report the incorporation of a microalgae-derived bio-binder into epoxy composites, promoting the integration of renewable resources into high-performance polymer matrices. Similarly, Souza et al. [Contribution 2] investigate biomass mastication as a pre-treatment method to improve the downstream processing of polyhydroxyalkanoates (PHA) synthesized by mixed microbial cultures. Complementing these experimental studies, the review by Getya and Gitsov [Contribution 3] offers an in-depth overview of hybrid polymer networks constructed from natural macromolecules such as cellulose, chitin, alginic acid, gellan gum, lignin, and their derivatives. Collectively, these contributions align with global efforts to transition toward biodegradable, bio-based polymer systems, as recently reviewed by Chauhan et al. [1].

This Special Issue also highlights *advances in functional and stimuli-responsive polymers.* Motoyanagi et al. [Contribution 4] demonstrate the synthesis of cyclic poly(N-isopropylacrylamide)s via Ring Expansion Reversible Addition–Fragmentation Chain Transfer (RE-RAFT) polymerization, enabling precise control of thermo-responsive behavior. Wilczewski et al. [Contribution 5] present a curcuminoid-functionalized graphene system that enhances dispersion within PVC-based nanocomposites, improving stability and uniformity. Braz et al. [Contribution 6] introduce hybrid nanoflowers anchored on polyamide microparticles for efficient cascade biocatalysis. This work notably employs wide- and small-angle X-ray scattering, illustrating the power of advanced characterization techniques using synchrotron radiation and spectroscopy for investigating soft matter that includes polymer-based materials—an approach recently emphasized by Takahara et al. [2]. Contributions 4 through 6 collectively underscore a growing focus on “intelligent” polymers with adaptable and multifunctional properties, consistent with the recent review by Jingcheng et al. [3].

Several studies in this Special Issue focus on *application-driven innovations* across critical technological sectors. Wang et al. [ Contribution 7] develop a solvent-free method for coating polyacrylonitrile onto Li_6_._4_La_3_Zr_1_._4_Ta_0_._6_O_12_ (LLZTO), a garnet-type solid-state electrolyte known for its high ionic conductivity and thermal stability in lithium batteries. Ranaiefar et al. [Contribution 8] combine additive manufacturing and machine learning to optimize the mechanical performance of carbon-reinforced ABS honeycomb composites, demonstrating the relevance of polymer-based materials in lightweight structural engineering. Kamo and Matsumoto [Contribution 9] investigate the role of pore size in epoxy monoliths designed for use as sheet-type adhesives—an area where polymer architecture critically influences bonding strength and durability. Contributions 7–9 reflect the broad utility of advanced polymer materials in energy, structural, and adhesive applications.

Additional contributions explore the design of *polymer composites with enhanced durability and tailored properties*. Smejda-Krzewicka et al. [Contribution 10] formulate butyl rubber nanocomposites filled with phyllosilicates, improving chemical resistance and barrier performance. Cao et al. [Contribution 11] describe a fluorinated flow modifier for boron nitrite/polyphenylene sulfide (BN/PPS) composites, yielding materials with high thermal conductivity, favorable dielectric behavior, and excellent processability via melt blending. These two studies reaffirm the importance of polymer systems with engineered molecular architectures and processing routes, enabling customized performance for a wide range of applications.

An unconventional yet notable contribution is the work by Wu et al. [Contribution 12], who apply silicone oil emulsion polymerization to the conservation of waterlogged wooden artifacts. This study demonstrates the expanding role of polymer science in *cultural heritage preservation.*

The two concluding review articles in this Special Issue extend its thematic reach into the *innovative polymer processing* domain. Mahmood et al. [Contribution 13] provide a comprehensive overview of polymer composites in 3D/4D printing, including discussions of materials, printing strategies, and structural design. Zhan et al. [Contribution 14] review advances in polymer-reinforced SiO_2_ aerogels, emphasizing improvements in both mechanical performance and fabrication processes.

Taken together, all contributions to this Special Issue reflect the decisive and multifaceted role of polymer-based materials in addressing current scientific and industrial challenges. This Special Issue also touches on several cross-cutting themes, including the application of machine learning in material design [Contribution 8], trade-offs between processability and performance (as seen in LLZTO-based systems and some adhesives [Contributions 7 and 9]), interface engineering in hybrid composites [Contributions 8–10], and the increasing use of advanced, non-destructive structural characterization [Contribution 6].

## 3. Editorial Perspective and Future Outlook

The fourteen papers contributed to this first edition of this Special Issue provide a representative snapshot of ongoing progress in polymer science and engineering. They effectively address critical challenges in polymer synthesis, material characterization, and the deployment of polymer-based systems in real-world applications. Strengths of this Special Issue include a clear emphasis on sustainability [Contributions 1–3], functional versatility [Contributions 4–6], and advanced processing techniques [Contributions 7–11], including also the 4D printing [Contribution 13], in which the 3D printed object changes its shape, properties, or functionality over time in response to external stimuli.

Nonetheless, a closer examination reveals opportunities for further exploration in future editions or new dedicated Special Issues. In particular, the integration of predictive modeling frameworks, as recently reviewed by Li et al. [4], along with the broader use of in situ and real-time characterization techniques (such as synchrotron radiation, neutron scattering, and reflectometry), and more comprehensive studies on degradation pathways [5] and lifecycle assessments [6], are essential for enabling the rational design, scalable production, and environmental responsibility of advanced polymer systems.

As of approximately one year after its closure, the first edition of this Special Issue has received over 50,000 views and 89 citations, reflecting substantial interest and engagement from the scientific community. A second edition of this Special Issue is currently open, with a submission deadline in April 2026, and is available on the following page: https://www.mdpi.com/journal/molecules/special_issues/UKD1605F0N. (accessed on 1 August 2025)

We hope that both editions of this Special Issue will continue to stimulate interdisciplinary research, strengthen collaboration between academia and industry, and support the advancement of next-generation polymer materials aligned with global sustainability and innovation goals.
